# Gender-related stress factors and emotional perception in migraine: a structured online questionnaire in migraine patients and controls

**DOI:** 10.1007/s10072-023-07152-6

**Published:** 2023-11-07

**Authors:** Marianna Delussi, Giulia Piraino, Simona Guerzoni, Flavia Lo Castro, Grazia Sances, Elena Guaschino, Gloria Vaghi, Licia Grazzi, Simona Sacco, Agnese Onofri, Giulia Paparella, Maria Pia Prudenzano, Maria Elena Roca, Adriana Fallacara, Sabina Cevoli, Giulia Pierangeli, Paola Sarchielli, Alessia Bellotti, Sara Invitto, Marina de Tommaso

**Affiliations:** 1 Department of Education, Psychology and Communication University of Bari Aldo Moro, Bari, Italy; 2 Department of Biological and Environmental Sciences and Technologies, University of Salento, Lecce, Italy; 3 Digital and Predictive Medicine, Pharmacology and Clinical Metabolic Toxicology‐Headache Center and Drug Abuse‐Laboratory of Clinical; Pharmacology and Pharmacogenomics, Department of Specialist Medicines, AOU Policlinico Di Modena, Modena, Italy; 4 Headache Science & Neurorehabilitation Center, IRCCS Mondino Foundation, Pavia, Italy; 5 Headache Center, Neuroalgology Dpt IRCCS Fondazione C Besta -Istituto Neurologico, Milan, Italy; 6 Department of Biotechnological and Applied Clinical Sciences, University of L’Aquila, L’Aquila, Italy; 7 Department of Translational Biomedicine and Neuroscience, Neurophysiopathology Unit, Headache and Chronic Pain Section, University of Bari Aldo Moro, Bari, Italy; 8 Headache Center, Amaducci Neurological Clinic, Policlinico General Hospital, Bari, Italy; 9 IRCCS Istituto Di Scienze Neurologiche Di Bologna, Bologna, Italy; 10 Headache Center, Neurological Clinic, Perugia, Italy

**Keywords:** Migraine, Gender, Stress, Emotions, Disease severity, Phenotype

## Abstract

**Background:**

While migraine is markedly prevalent in women, gender-related phenotype differences were rarely assessed. For this reason, we investigated, through a multicenter observational cross-sectional study, based on an online questionnaire, gender-related differences in stress factors, emotions, and pain perception in migraine patients and controls and their impact on migraine severity.

**Methods:**

The study was designed as an online questionnaire. The link was emailed to healthy subjects (C) and migraine patients (MIG) (age 18–75, education ≥ 13 years) recruited during the first visit in 8 Italian Headache Centers adhering to Italian Society for Headache Study (SISC). The questionnaire included personal/social/work information, the Perceived Stress Scale, the Romance Quality Scale, the Emotion Regulation Questionnaire, the Beck Anxiety Inventory, the Body Perception Questionnaire, the pain perception, and a self-assessment of migraine severity in the last 3 months.

**Results:**

202 MIG and 202 C completed the survey. Independently from gender, migraine was characterized by higher pain sensitivity and more severe partner relationships. The female gender, in MIG, exhibited higher anxiety scores, body awareness, and reduced emotional suppression. Body awareness and emotional suppression were discriminating factors between genders in control and migraine groups without relevant influence on disease features. Perceived perception of migraine severity was similar between genders.

**Conclusion:**

Gender-related emotional and stress factors did not contribute to delineate a distinct phenotype in migraine men and women. The possible impact of emotional and stress factors characterizing genders could be considered for a single case–tailored therapeutic approach.

**Supplementary information:**

The online version contains supplementary material available at 10.1007/s10072-023-07152-6.

## Introduction

Migraine is highly prevalent in the general population, with a clear predominance in female gender. [1]. Genetic, hormonal, and environmental factors could contribute to the prevalence of migraine in females [2].

Personalized medicine attempts to foresee the outcome of diseases based on specific risk factors. At the same time, several studies described the influence of female sexual hormones on migraine [3]. Gender-related differences in migraine phenotype were rarely assessed due to the low number of males generally included in the study groups [4]. Profound differences in neuronal circuits and neurotransmitters in pain processing characterize the two sexes [5]. Pain threshold is generally reported as reduced in female animal models and humans, who exhibited more efficient resilience to continuous pain [6].

Perceived stress and negative social and familiar context could facilitate the expression of migraine in females [4, 7]. A sizeable Canadian population study demonstrated a high synergism between the female gender and stress on the risk of migraine [8]. An online survey based on a large cohort of migraine patients indicated that women more than men reported stress as a migraine trigger [9], while in another study, the effect of stress on migraine severity did not appear to be linked to gender [10]. Another recent study stated that among women, work conditions considered as stress cause, and in particular, shift work, were associated with higher odds of migraine but not tension-type headache [11].

In the present study, conducted in 8 headache centers adhering to the Italian Society for Headache Study (SISC), we built an online questionnaire on perceived stress, stress factors in the family context and workplaces, and subjective perception of headache severity to be proposed to patients with the diagnosis of migraine and healthy subjects, balanced for sex and age. The study’s central hypothesis was that gender-related differences in stress perception could impact migraine phenotype. With this aim, we explored gender-related differences in family relationships, women’s violence history, perceived stress, and emotional regulation, in migraine patients and controls. Then, we aimed to understand if migraine features differed between genders, and how gender-related dissimilarities in stress and emotional factors impacted migraine severity. We also explored the effect of age on the considered variables to understand if the menopausal age range and related hormonal changes could have a role in stress perception and migraine features.

## Method

The study was conceived as a detailed online questionnaire, accessible via the Internet by a link, and sent by email to migraine patients who first visit eight tertiary headache centers adhering to the Italian Headache Society (SISC). Criteria for the study proposal were a diagnosis of migraine (with or without aura and chronic migraine), according to IHS criteria [12]. The exclusion criteria for the selection of study groups were age < 20 and > 70; chronic neurologic, psychiatric, and general medical diseases (excluded migraine); education < 13 years (at least high school diploma). The survey was anonymous. Age was also indicated in terms of 10 years’ ranges. The total time requested to complete the questionnaire varied from 15 to 20 min.

The same link was proposed for a sample of healthy subjects selected from universities and hospital staff in the eight centers.

The Local Ethic Committee of Bari Policlinico General Hospital approved the study, pending the deletion of individual characteristics with potential violation of privacy policy, including specific comorbidities and therapies. The informed consent document preceded the questionnaire, and the participant signature served to proceed with the completion of the form. The study was conformed with the World Medical Association Declaration of Helsinki published on the website of the Journal of American Medical Association.

### Methodology

#### Scales and questionnaires

The questionnaire was divided into three sections: the first included personal and social data, with work situation; the second section included behavioral questionnaires to investigate the cognitive, emotional, relational, and perceptual functioning of the participants. The third section was exclusive for migraine patients, considering scores of migraine severity in the last 3 months.

The second session included the following constructs: partnership violence, through a dichotomous item (Supplementary Material 2,3), body perception (i.e., Body Perception Questionnaire) [13], perceived stress (i.e., Perceived Stress Scale PSS) [14], anxiety symptomatology (i.e., Beck Anxiety Inventory BAI) [15], emotional regulation (i.e., Emotional Regulation Questionnaire ERQ and Emotional Suppression ERS) [16], quality of the relationship (i.e., Romance Qualities Scale RQS) [17].

Pain sensation through a question about the maximal pain suffered in the last 3 months, evaluated on a numerical scale from 0 to 10.

A detailed description of tests is reported in the Supplementary material (Supplementary material 1).

The third section assessed migraine features: this section included specific items for a self-evaluation of migraine intensity during the previous 3 months, evaluated with a numerical scale from 0 to 10, and a migraine disability assessment related to difficulty in work and social relationships. We choose not to use validated disability questionnaires, such as MIDAS [18] and HIT-6 [19], for possible difficulty in their online self-administration.

(Supplementary materials 2 and 3).

### Statistical analysis

For statistical analyses, SPSS version 21 was used. The descriptive analyses explored the distribution of demographic and social data of the participants.

After applying the Levene test for equality of variance, we used the two-way ANOVA analysis for single variables related to perception, emotional functioning, and relationship quality, considering group (migraine-MIG vs. healthy subjects—HS) and gender as factors. Statistical significance was set at *p* < 0.05.

The dichotomous variables were evaluated using the chi square test.

For migraine, we performed a MANOVA analysis with gender, dichotomous variables such as the history of violence, work situation, and disease severity scores as variables. Those continuous variables, significantly different between genders, were introduced as predictors for migraine severity scores, using a linear multi-regression analysis, *F* probability settled at < 0.05.

To investigate which variable better discriminated between men and women with migraine, we introduced a step-way discriminant analysis with leave on out method, introducing those variables which were dissimilar between genders in the entire database.

## Results

We sent the questionnaires to a total of 500 migraine patients and 500 controls, preliminarily selected for exclusion-inclusion criteria and matched for sex and age. No woman was pregnant at the time of the questionnaire mailing. Two hundred and two migraine patients completed the questionnaires, while the remainder did not respond at all or sent not completed responses. We thus selected the first 202 normal questionnaires, among the 255 globally completed by controls.

### Demographic and personal data

Gender was equally distributed between patients and controls (126 females and 76 males in migraine group, 137 females and 65 males in control group, chi square 1.31; *p* 1.25). Age was similar between groups (chi square 1.29; *p* 1.22). Females prevailed in all the age ranges in both groups (Table 1). The 63.9% of migraine patients and 40.1% of controls had full time stable work activity, 26.7% of migraine patients and 32.2 of controls were unemployed, 9.4% migraine patients, and 25.7 of controls had partial time temporary work, so stable work prevailed in migraine group (chi square 149; *p* < 0.0001). Taking into consideration gender in the total of cases, stable work prevailed in men (66.7 males, 44.1 females, chi square 21.66; *p* < 0.001). The prevalence of men with stable occupation was present in migraine and control groups as well.

**Table 1:** distribution of migraine patients and controls in the age ranges, distinguished for gender

Age range (years)	Migraine	Controls	Total
20–30	16 f 15 m	22 f 23 m	76
31–40	31 f 10 m	38 f 13 m	82
41–50	36 f 28 m	35 f 10 m	124
51–60	33 f 18 m	32 f 15 m	95
61–70	10 f 5 m	10 f 4 m	27
	202	202	404

#### Behavioral questionnaires

### Violence

Cases reporting episodes of suffered violence, were equally distributed between migraine (29.2%) and control groups (32.7%). Women with previous violence history, prevailed in the whole of cases (35% females, 23.1% males; chi square 5.75; *p* 0.016), and in the migraine groups separately considered, but not in the control group. In fact, in control group, the 33.6% of women and 30.8% of men reported a history of violence of different type (chi square 0.15; *p* 0.19). In migraine group, women with violence history prevailed on men (36.5 in females, 17.1% in males; chi square 8.63; *p* 0.003).

**Fig. 1:**
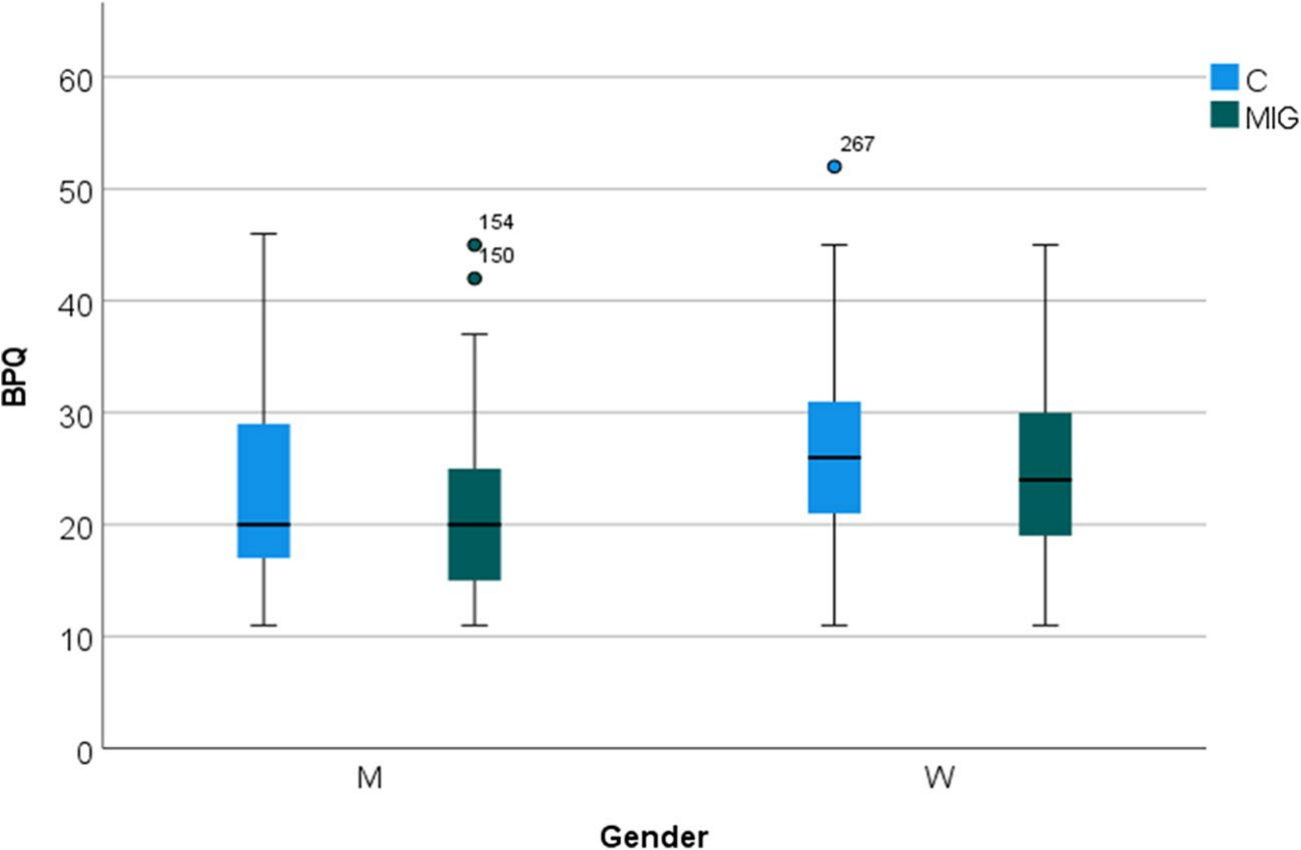
Box blot of BPQ (body perception questionnaires in migraine patients (MIG) and controls (C), divided according to gender men (M) and women (W). There was a slight reduction of body awareness in migraine patients (*p* < 0.05). M showed reduced expression of body perception, independently from migraine diagnosis (*p* < 0.001)

Perceptual functioning. Body perception scores indicated a slight reduction of body awareness in migraine patients (group as factor, *F* 4.91; *p* 0.027). Men showed reduced expression of body perception (gender as factor, *F* 47.67; *p* < 0.001), independently from migraine diagnosis (interaction gender × group, *F* 0.12 ns) (Fig. 1).

### Perceived stress (PSS)

**Fig. 2:**
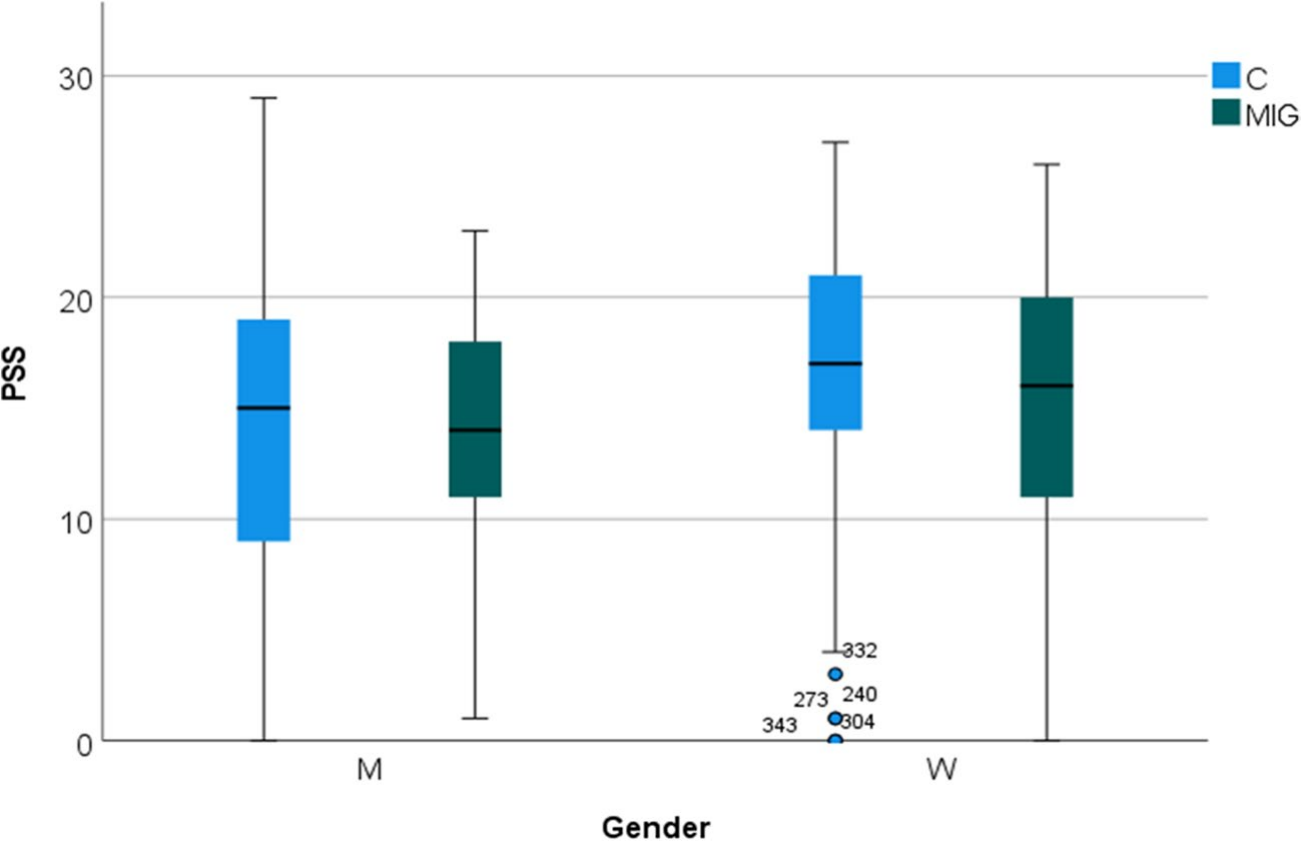
Box blot of PSS scores (perceived stress) in migraine patients (MIG) and controls (C), divided according to gender men (M) and women (W). Scores were higher in W, independently from diagnosis of migraine (*p* < 0.01)

Perceived stress was similar between migraine and controls (group as factor, *F* 2.16 n.s.), but showed higher scores in women, independently from diagnosis of migraine (gender as factor, *F* 9.38; *p* 0.002; interaction gender × group, *F* 0.7 n.s. (Fig. 2).

### Perceived anxiety (BAI)

It was similar between migraine patients and controls (migraine women 16.9 ± 4.5; men 12.5 ± 5.5; controls women 17.57 ± 3.8; men 13.83 ± 4.5 men group as factor, *F* 0.6 n.s.) but scores were significantly increased in women (gender as factor, *F* 9.91; *p* 0.002), independently from migraine diagnosis (gender × group, *F* 0.056 n.s.)

### Pain sensitivity

Pain sensitivity scores were higher in migraine patients, in respect to controls (migraine women 8.31 ± 1.1 men 8.12 ± 1.2; healthy women 6.62 ± 1.3; men 5.53 ± 1.4 group as factor, *F* 96.92; *p* < 0.0001). While women exhibited pain scores mildly increased in respect to men, this difference was not significant (gender as factor, *F* 1.94 n.s.; interaction gender × group, *F* 0.38 n.s.).

**Fig. 3:**
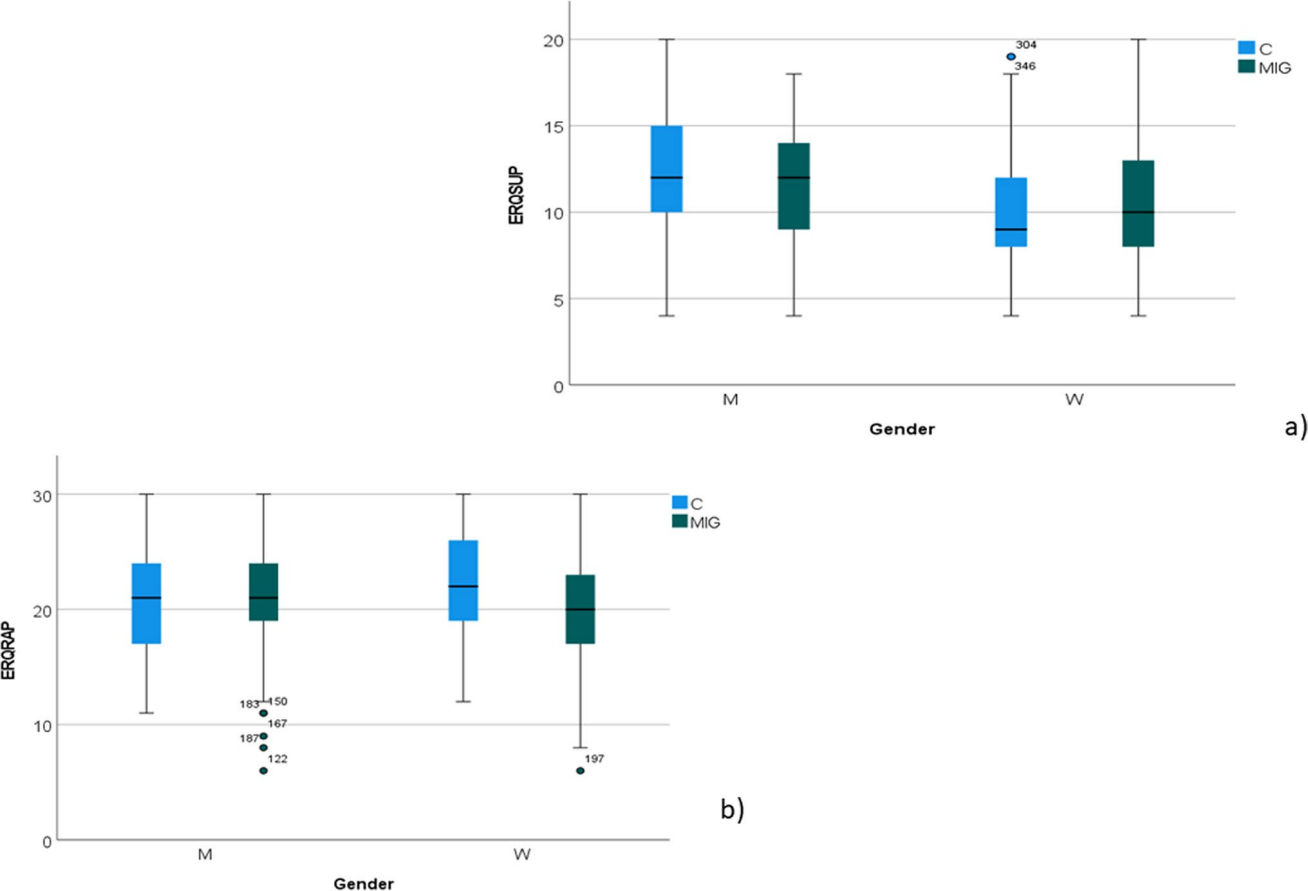
**a** Box blot of emotional regulation scores in migraine patients (MIG) and controls (C), divided according to gender men (M) and women (W). Scores were reduced in migraine patients (*p* < 0.01), independently from gender. **b** Emotional suppression scores (ERS) scores were significantly higher in M (*p* < 0.001). M with migraine showed lower ERS scores (interaction diagnosis × gender, *p* < 0.05)

Emotional functioning. Emotional regulation (ERQ) was reduced in migraine patients as compared with controls (group as factor, *F* 9.29; *p* 0,002). The ANOVA with gender as factor (*F* 0.6 n.s.) as well as the interaction with group (gender × group, *F* 3.06; *p* 0.08) were not significant (Fig. 3a).

Emotional suppression (ERS) was similar between patients and controls (group as factor, *F* 0.2, n.s.), but was clearly dissimilar between genders (gender as factor *F* 15.34; *p* < 0.001), as emotional suppression prevailed in men. However, men with migraine had lower ERS scores in respect to control men (interaction gender × group, *F* 4.31; *p* 0.038) (Fig. 3b).

### Romantic relationship

**Fig. 4:**
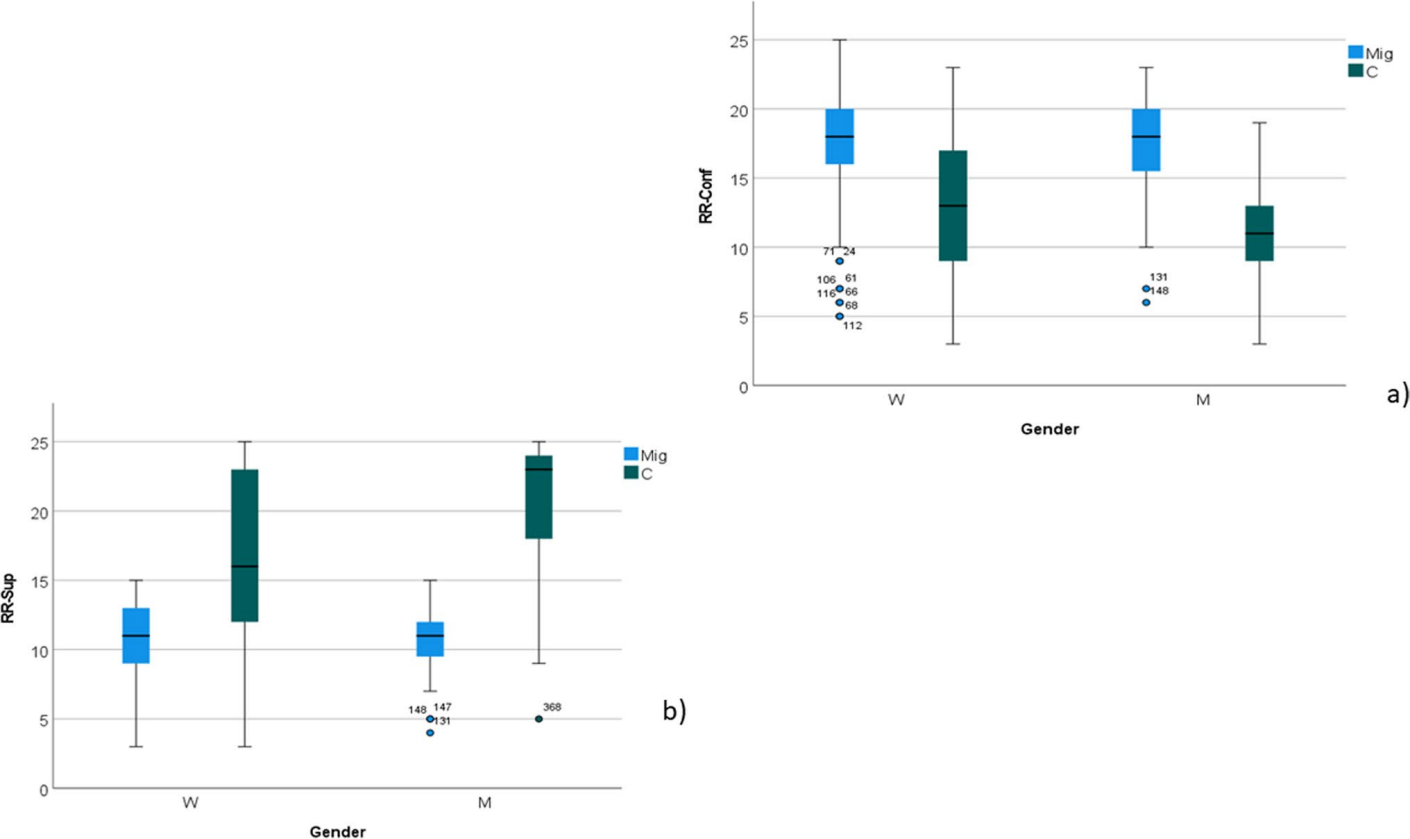
**a** Box blot of romantic relationships–perception of conflict scores in migraine patients (MIG) and controls (C), divided according to gender men (M) and women (W). The perception of conflict within the relationship was higher in MIG (*p* < 0.001) and in W (*p* < 0.01), while the interaction between gender and diagnosis was not significant; **b** romantic relationships—the scores of perception of emotional support were reduced in W (*p* < 0.001) and in MIG (*p* < 0.001). The interaction between gender and diagnosis was also significant (*p* < 0.001), as migraine diagnosis implied smaller differences between genders

Twenty-five participants with migraine and 20 controls declared no current romantic relationship, or preferred not to complete the questionnaire. The perception of conflict within the couple was higher in migraine group (group as factor, *F* 108; *p* < 0.001) and in women (gender as factor, *F* 7.29; *p* 0.007), while the interaction between gender and group was not significant (*F* 3.090; *p* 0.080) (Fig. 4a).

The perception of emotional support within the couple, was reduced in women (gender as factor, *F* 13.18; *p* < 0.001), and in migraine group (group as factor, *F* 19.5; *p* < 0.001). The interaction between gender and group was also significant (*F* 14.36; *p* < 0.001), as migraine diagnosis implied smaller differences between genders (Fig. 4b).

The other romantic relationship scores were similar between men and women, as well as between migraine patients and controls.

### Features of migraine

The MANOVA analysis including the subjective judgment of headache intensity in the last 3 months and the impression of disability linked with headache did not show significant difference between women and men with migraine (gender as factor, *F* 1.42; *p* 0.2). The only variable which was significantly different between women and men was the subjective impression of headache intensity, evaluated on average in the last 3 months (migraine women 6.03 ± 1.4; men 5.43 ± 1.6, *F* 5.93; *p* 0.016).

The number of days with migraine disability was also similar between genders (88 patients with 0–6 days, 44 patients with 7–10 days, 67 patients with 15–30 days; chi square for gender, 2.78; *p* 0.24).

Features of migraine were also similar between genders, taking into consideration working status (MANOVA working for gender, *F*-Roy square 0.87; *p* 0.51). The history of violence determined more severe features of migraine, independently from gender (*F*-Roy square 2.65; *p* 0.017; history of violence × gender, *F* 0.83; *p* 0.56). Considering single variables, increased disability in social activity and domestic work approached the statistical significance in patients with reported history of violence (respectively *F* 3.70; *p* 0.059; *F* 3.55; *p* 0.066).

The multi-regression analysis considering the subjective perception of headache intensity as variable and emotional suppression, perceived stress, body perception, perceived anxiety and romantic relationships scores (perception of conflict and support within the couple) was not significant (*r* 0.26; *r* square 0.072; ANOVA *F* 1.81; *p* 0.1). Reduced body perception and increased emotional suppression were the factors discriminating men from women with migraine (Wilk’s lambda 0.912; chi square 13.25; *p* 0.001), with an accuracy of 62.3% of cases correctly classified.

### Effect of age

Body perception was lower in the 61–70 age range (effect of age, *F* 2.45; *p* 0.046; Bonferroni test 61–70 age range vs. the remainder age groups, *p* < 0.05), with prevalence in men (effect of age × gender, *F* 3.11; *p* 0.011). There was no significant effect of migraine diagnosis.

Perceived stress was also reduced in the 61–70 age range (*F* 3.18; *p* 0.014; Bonferroni test 61–70 age range vs. 20–30, 31–40, 41–50, *p* < 0.05), independently from gender and migraine diagnosis.

Perceived anxiety was affected by the interaction age × gender (*F* 3.85; *p* 0.004), as women in the 20–30 age range had higher scores than those in the 61–70 age group (Bonferroni *p* < 0.01). Migraine diagnosis had no effect.

Age did not influence pain sensitivity, emotional regulation, and suppression.

Age had no effect on subjective perception of headache intensity within migraine group.

## Discussion

In this study, we explored possible gender-related differences among a population of migraine patients compared to controls, taking into consideration factors such as perceived stress, emotional regulation, and social and familial conflicts, eventually impacting disease features.

We found that, independently from gender, migraine was characterized by higher pain sensitivity and less emotional regulation to controls. Both males and females with migraine declared more severe conflicts with their partners to controls. The female gender, independently from diagnosis of migraine, exhibited higher anxiety scores, higher body awareness, and reduction emotional suppression. Gender did not influence the subjective judgment of migraine severity, a part from the increased perception of migraine intensity. Factors discriminating genders in the migraine group, body awareness, and emotional suppression were the same differentiating genders in the control group.

Older men demonstrated lower scores of perceived body perception and perceived anxiety, independently from migraine diagnosis.

In the next paragraph, single results will be discussed.

### History of violence

Females with migraine, reported higher prevalence of episodes of violence compared to males, while the impact of familiar violence on migraine disability was present in both genders. History of abuse and violence has been described in migraine patients, associated to more severe disease and pain hypersensitivity [20]. Another study conducted in pregnant women found that intimate partner violence was associated to higher risk for migraine [21]. We did not evaluate the type of violence, physical, or emotional, for the limited number of cases, but the general report of episodes of abuse should be evaluated in clinical practice, mainly in migraine females.

### Working activities

We found a prevalence of stable employment in the migraine group, which is quite a surprising result. A study aiming to validate a scale for work productivity and activity impairment in migraineurs found a high presence of patients with work-related activity impairment and work productivity loss, which is more compatible with the employee than the freelance situation. While employees prevailed in males with migraine, this aspect did not have an impact on the subjective impression of disease severity, though unemployment is a general cause of psychiatric disturbances among females [22]. Moreover, in a recent study conducted among migraine patients during COVID-19 outbreak, we observed that the working activities reduction and the “stay at home” prescription improved migraine severity, so in this sense the housewife work could not have an unfavorable impact on migraine severity [23].

### Perceptual functioning

Body awareness was reduced in men in the whole of the participants, and it was one of the two factors discriminating gender within migraine group. Women tend to show an advantage over men in the processing and recognition of their own and other’s emotions [24]. Women also self-report more engagement by their own emotions, demonstrating higher scores for emotional self-awareness [25]. We found a mild reduction of body awareness in migraine. Reduced body awareness has been described in chronic pain, with an uncertain Impact on disease severity [26]. In our migraine group, while males displayed reduced body perception, they did not display a worse perception of disease features, in accord with the unclear impact of this factor on pain severity. Age-influenced body perception, being reduced in older men, for a possible complex sexual hormonal interaction, deserving further analysis.

### Perceived stress

Women seemed in general more sensitive to stress, independently from the diagnosis of migraine. An extensive body of animal and human studies literature described biochemical sex differences in response to stress [27], based on neuroendocrine and anatomical factors [28]. Moreover, perceived stress did not prevail in women with migraine compared to controls, nor we found gender-related differences in the severity of migraine. This was a quite unexpected finding, as the susceptibility of migraine patients to stress is well-known [29]. However, the direct relationship between perceived stress and the incidence of migraine remains unclear, as the occurrence of migraine could be multifactorial and not directly connected to a generic maladaptive response to stress [30].

Older subjects displayed reduced stress questionnaire scores, independently from gender, which could be attributed to possible age-related lifestyle changes.

### Perceived anxiety

This item also differed between the two genders, but not between migraine and controls. The prevalence of anxiety in women has been referred to multiple factors, as hormonal, genetic, and psychosocial [31]. Hormonal and psychosocial factors could also justify the prevalence of anxiety found in younger women. Anxiety disorder is one of the most represented comorbidities in the migraine population [32], so we can assume that the items we used were not sensitive in identifying relevant symptoms in migraine patients. However, the Beck Anxiety Inventory is a reliable indicator of anxious symptoms in migraine [33] and the general population.

Moreover, the control population was selected among university and hospital staff, which could be a bias due to the frequent representation of anxiety among students and healthcare workers [34]. On average, the BAI scores indicated mild to moderate anxiety in both women and men with migraine. This element was not associated with the increased perception of migraine intensity we found in migraine women.

### Pain sensitivity

This item consisted of the self-evaluation of the intensity of worst pain perceived in the last 3 months, which, as expected, prevailed in migraine patients [35]. We selected controls for the absence of chronic neurological and medical diseases, including any form of chronic pain, so the question was addressed to establish the maximal sensation proven during any type of noxious sensation recently occurred. We can assume that several control cases could not remember the pain in the last 3 months, so the difference we found with migraine patients is highly conceivable. Pain threshold is generally reduced in females, as estrogens and androgens have different modulatory effects on pain control [36]. However, the complexity of neurotransmission differences between sexes should not enable to attribute a fixed pattern of pain hypersensitivity to the female gender, so a simple question about the maximal pain sensation perceived could not capture subtle gender-related differences.

### Emotional regulation and emotional suppression

The term “emotion regulation” defines how individuals try to control or change their behavior due to prevailing emotions [37]. Females and males with migraine showed emotional dysregulation, without relevant differences between genders. The aspect which characterized the masculine profile was emotional suppression. Emotional suppression refers to the voluntary repression of behavior during emotional arousal. The capacity to inhibit dynamic behavior is well-known in males [37], and this aspect also discriminates genders within the migraine group. However, increased emotional suppression seemed not to influence migraine features, as subjectively reported by women and men with migraine.

### Romantic relationships

Increased conflicts and reduced emotive support within the couple seemed to characterize migraine population and female gender. Several studies assessed the negative impact of migraine on family life, especially on spouses and children [38]. A large prospective, longitudinal, web-based survey study assessed the perceived impact of migraine on family relationships and found that migraine negatively impact romantic and family relationships, with more significant burden in chronic migraine. However, the same study did not show substantial differences for familiar conflicts between genders [39].

### Features of migraine

Women and men with migraine, similarly judged their migraine, except for increased perception of migraine intensity in the last 3 months. Migraine disability was quite similar in women and men. Pain sensitivity is in general enhanced in female gender [6], but this aspect did not impact migraine disability as expressed with the questionnaire. We explored if the variables significantly different between gender in the whole of participants, as body perception, emotional suppression, and familiar conflicts, influenced pain perception. The result was negative, so the increased pain sensitivity in women with migraine does not seem to be associated to specific gender-related emotional and perceptional factors. This confirms the complexity of pain regulation and the difficulty in individuate a specific profile related to genders within migraine patients [36]. In fact, body perception and emotional suppression clearly characterized genders in the migraine group, but these characteristics did not impact migraine features. Working status and history of violence did not influence migraine severity in a different mode in women and men.

## Study limitations

The study was limited by the low number of cases completing the survey, probably for its length and complexity. We proposed standardized tests for reliability purposes, but in many cases the self-administration could request some assistance. In order to preserve the anonymity of patients, in consideration of the intimate content of some items, we deleted any demographic or clinical detail, which in any case could have relevance for migraine profile, as duration of illness or previous treatments. In order to previously ascertain the inclusion–exclusion criteria, the control sample was selected among university and hospital staff, which could not be exemplificative of general population. Finally, the questionnaire did not include specific items about sexual orientation, a part from a generic choice among female, male, and a generic “other” gender. Further analysis could be dedicated to this important issue.

## General remarks

Summarizing, the present questionnaire proposed to a small sample of migraine patients and healthy controls failed to identify gender-related emotional and stress factors with relevant incidence on migraine features and severity. The numerous aspects differentiating the two genders, as perceived stress, emotional suppression, body awareness, history of violence, and romantic conflicts, did not imply different migraine profiles in women and men. Some critical aspects emerged from these data, as the prevalence of history of violence and problematic romantic relationships in migraine, independently from gender. The general consideration of the negative impact of these variables on migraine is outside the aims of this study, but they could be worthy of consideration in further studies in large migraine cohort, as well as in clinical practice.

Migraine phenotype, seems to be similar between genders, even though women and men are deeply different as regard to emotion expression and stress perception, that in theory could have an impact on disease severity. We can assume that the genetic and hormonal influences on neuronal circuits cooperate in identifying a migraine phenotype which is more or less similar across genders. However, in a prospective of precision medicine, the possible impact of emotional and stress factors clearly characterizing the genders could be taken into consideration for single case–tailored therapeutic approach.

### Supplementary Information

Below is the link to the electronic supplementary material.

Supplementary file1 (PDF 2366 KB)

Supplementary file2 (PDF 1754 KB)

Supplementary file3 (DOCX 12 KB)

## Data Availability

Dataset is available on request.
