# Supplementary material for: Gender-related stress factors and emotional perception in migraine: a structured online questionnaire in migraine patients and controls
Source: Neurol Sci. 2023 Nov 7;45(4):1645–54. doi: 10.1007/s10072-023-07152-6 (PMC10942877; doi:10.1007/s10072-023-07152-6)
Supplement: Supplementary file 1 — Supplementary file1 (PDF 2366 KB) [file 10072_2023_7152_MOESM1_ESM.pdf]

# Gender influences in chronic pain - migraine

Dear Madam/s,

we are conducting research on the influence of work and family stress on your chronic pain pathology. This study is coordinated by the University of Bari, AOU Policlinico. The scientific manager of the study is Prof. Marina de Tommaso

Participation consists in filling out some questionnaires, it will steal about ten minutes of your time.

Please read the following carefully.
The University Hospital Policlinico of Bari as Data Controller, and the internal manager of the treatment, will process your personal data, in particular those relating to health and, only to the extent that they are indispensable in relation to the objective of the study , other data concerning its origin, and genetic data, exclusively in function of carrying out the study.
The person authorized to process data under the authority of the Data Controller is Prof. Marina de Tommaso at the Amaducci Neurological Clinic/Neurophysiopathology

1. PURPOSE OF THE TREATMENT
Scientific and statistical research aimed at protecting the health of the community in the medical, biomedical and epidemiological fields.
2. NATURE OF THE DATA (which data will be collected and processed)
The data collected and processed will

be: - personal data: any information concerning a natural person, including an identification number regardless of the medium used (paper, electronic, etc.); - sensitive data, for

example those concerning the state of health and origin

3. LEGAL BASIS OF TREATMENT The

personal data you provide for the purposes referred to in point 1 will be processed exclusively on the basis of your express consent, which you may revoke at any time. Any revocation will have value only for the future, the treatment carried out up to that moment remaining valid. The data collected up to the moment of the revocation will be included in the study, the subsequent deleted.
4. NATURE AND CONSEQUENCES OF THE PROVISION OF

DATA The provision of your data, absolutely optional, is however necessary for participation in the study or, in any case, for the pursuit of the aforementioned purposes. Failure to provide data may prevent participation in the study, in whole or in part. Participation in the study takes place on a voluntary basis, therefore, failure to consent to the processing of data for this purpose does not affect the right to use the other medical-health services provided by the Centre.
5. DATA PROCESSING METHODS The

collection, recording, storage and modification of personal data will take place using IT tools with logic strictly related to the purposes referred to in point 1. The data will be processed by applying the appropriate measures of

security pursuant to articles 32 and following of EU Regulation 2016/679 and, this, both from an organizational and from a technological point of view. Your personal data will be collected with the use of a code that will not allow your direct identification, because the documentation that allows you to recover your identity will be kept and kept by the Investigator, separately from the documents, and will only be accessible to subjects specifically authorized him.

6. COMMUNICATION AND DIFFUSION

Your personal data cannot be disclosed, except in aggregate form and, therefore, in an absolutely anonymous way. Your data may be made available to subjects, including external ones, expressly designated as data processors, in pseudo-anonymised electronic form. (i.e. not associated with its name, but with a code).

7. DURATION OF THE TREATMENT

The data you provide and subsequently processed will be kept for a period of time not exceeding that necessary to achieve the purposes for which they were collected and processed and, therefore, for a period of 36 months.

8. EXERCISE OF RIGHTS

You may exercise the rights of the interested party at any time, as governed by articles 13 and following of EU Regulation 2016/679. In any case, you may at any time, and in the cases provided for by law, request access, rectification and deletion of data, their portability to another holder, as well as request the limitation of treatment and oppose the same. You may also revoke your consent, thus being able to interrupt your participation in the study at any time and without providing any justification; in this case, no further data concerning you will be collected, without prejudice to the use of any data already collected as indicated in point 3. To exercise these rights, you can submit a request, using the method you deem most appropriate, directly to the data controller, the manager or even the staff. You can also write to the Data Protection Officers designated by the owner himself, who can be contacted via the following email addresses: marina.detommaso@uniba.it; gianni.lucatorto@policlinico.ba.it

You also have the right to lodge a complaint with the supervisory authority of your country of residence. In the event that you are resident in Italy, the authority to which to submit the complaint is the Data Protection Authority, for whose instructions you can connect to the link https://www.garanteprivacy.it/web/guest/home/docweb/-/docwebdisplay/docweb/4535524

8. Technical cookies

No personal user data is acquired by the site for this purpose. Cookies are not used for information of a personal nature, nor are persistent cookies of any kind or user tracking systems used. The use of session cookies (which are not stored permanently on the user's computer and disappear when the browser is closed) is strictly limited to the transmission of session identifiers necessary to allow safe and efficient exploration of the site. The session cookies used on this site avoid the use of other IT techniques potentially prejudicial to the confidentiality of users' browsing and do not allow the acquisition of personal identification data of the user.

9. Third-party cookies

Furthermore, cookies from third parties will not be used.

10. For what purposes do we process your data?

The processing of your personal and "particular" data (such as, for example, those suitable for revealing the person's state of health and/or sex life, racial or ethnic origin, religious beliefs and genetic data) will be carried out exclusively for the purposes set out below:

10.1. study and research purposes

11. Legal bases that legitimize the treatment The treatment of the collected data is legitimized by the art. of the GDPR, as explained below for each specific purpose, referred to in the following points of this information:

12. Who do we communicate your data to?
The data will not be disclosed to anyone

The data being processed, as suitable for revealing the state of health, will not be disclosed.

13. Service delivery methods The services are provided through the use of: WEBSurvey;

14. How long do we keep your data?
All personal data acquired through the Google Forms will be processed for as long as the evaluation period lasts. At the end of this period, the data will be kept for 01 year and subsequently cancelled.

15. What obligations do we have towards you?
We have an obligation to respond to your requests and, specifically, to allow you to exercise your rights as set out below:

a) Access: know what data we process, how and why we process them b)
Rectification: correct inaccurate personal data, where applicable c)
Cancellation (right to be forgotten) d)
Limitation of processing: feasible only for some specific situations e) Receive communication in case of rectification, cancellation, limitation f) Right to data portability: not applicable for treatments carried out on the basis of a law or regulation and for "derived" data such as, for example, assessments concerning the state of health (reports and health documentation in general) g) Right to object: the interested party has the right to object at any time to the processing for purposes other than those directly or indirectly connected to hospitalization for purposes of diagnosis, assistance and health therapy, without prejudice the existence of legitimate reasons that prevail over the interests, rights and fundamental freedoms of the interested party or for the assessment, exercise or defense of a right in court h) Right to withdraw consent: applicable only to processing carried out on the basis of the release of the consent, however remaining valid for the treatments carried out prior to the revocation
i) Right to lodge a complaint with a Supervisory Authority: if you believe that you have not received adequate answers to your requests, you can contact the Authority

Privacy Guarantor of the state in which you reside or work or propose an appeal before the judicial authority

16. Are you obliged to provide your data?

Apart from that specified for navigation data, the user is free to provide personal data or data indicated in telephone contacts and during clinical management.

Failure to provide them may make it impossible to obtain what is requested Once the user has decided to use the services offered by the WEBAPP, the provision of data is necessary for the correct provision of the services themselves and the failure to transmit them or a their partial or inexact transmission will make it impossible for the user to use these services. The processing of the aforementioned data is carried out for the aforementioned purposes, by the IT systems with which the WEBAPP cooperates, in compliance with the provisions of article 12 of the RGDP by means of electronic or in any case automated tools, in compliance with the rules of confidentiality and safety measures required by law and/or by regulatory legislation.

17. Where does your data come from?

The personal data necessary for the correct implementation of the activities directly or indirectly connected to the health services provided on an outpatient basis are provided directly by the interested party.

18. Complaint to the supervisory authority.

Pursuant to art. 77 of EU Regulation 2016/679, we remind you that you have the right to lodge a complaint with the Supervisory Authority (Guarantor for the protection of personal data), in the event that you believe that the processing that concerns you violates the provisions of the same Regulation .

19. Existence of automated decision-making processes in the treatment.

It is specified that for the processing of the above data there is NO type of automated decision-making process, pursuant to art. 22 of EU Regulation 2016/679 20. There is no transfer of personal data outside the EU

Who to contact and how to exercise your rights?

To assert your rights as provided for in CHAPTER III of Regulation 2016/679 (briefly explained in point 7) you can contact the Personal Data Protection Officer of the AOU Policlinico di Bari ,

Responsible for the research project to whom you can ask for further and possible clarifications:

Prof. Marina de Tommaso marina.detommaso@uniba.it

If in accordance with the above, you can click on YES

* Indicates a mandatory question

Default title

Untitled section

Untitled section

Machine Translated by Google


1.

*Select all applicable entries.*

☐ YES

## Personal characteristics

In this section you will find questions aimed at collecting some information of a personal nature and connected to your history.

As you read in the previous section, all data will be treated anonymously.

2. gender *

*Mark only one oval.*

- Man
- Woman
- Other

3. How old are you *

*Mark only one oval.*

- between 20 and 30
- between 31 and 40
- between 41 and 50
- between 51 and 60
- between 61 and 70
-
-

4. Degree *

*Mark only one oval.*

- ( ) I
- ( )
- ( ) high school diploma
- ( ) degree
- ( ) post graduate training
- ( )

5. Marital status *

*Mark only one oval.*

- ( ) Maiden
- ( ) Married
- ( ) Separate
- ( ) Widow

6. In which region do you live? *

*Mark only one oval.*

- Abruzzo
- Basilicata
- Calabria
- Campania
- Emilia Romagna
- Friuli Venezia Giulia
- Lazio
- Liguria
- Lombardy
- Brands
- Molise
- Piedmont
- Puglia
- Sardinia
- Sicily
- Tuscany
- Trentino Alto Adige
- Umbria
- Val d'Aosta
- Veneto

7. Current work situation *

*Mark only one oval.*

- unemployed
- occasional employment (part-time / on call)
- regular employment (full - time / full time)

8. Are you a smoker/smoker? *

*Mark only one oval.*

- YES
- NO

9. Do you have chronic conditions other than migraine? *

*Mark only one oval.*

- YES
- NO

10. Do you think you have ever been subjected to one or more of the following types of violence? *
(may indicate multiple answers)

*Mark only one oval.*

- Physics
- Psychological
- Economical
- Sexual
- Stalking
- Mobbing
- Threats
- none of the above
- Other: __________

11. Are you currently in a relationship? If you answer no, you can skip the section 4 *

*Mark only one oval.*

- Yes
- No

Machine Translated by Google


12. How long have you been in a couple relationship?

*Mark only one oval.*

- Less than a year
- From one to three years
- More than three years

13. Do you have children? *

*Mark only one oval.*

- Yes minors
- Yes, adults
- Yes, both minors and adults
- NO

14. Do your children live with you? *

*Mark only one oval.*

- Yes
- No
- I have no children

15. Do you have other family members besides your children that you take care of? *

*Mark only one oval.*

- Yes
- No

18. To feel better (happy/content/uplifted/in a good mood) I try to look at things from a different perspective *

*Mark only one oval.*

Not at all agree

1

2

3

4

5

Totally agree

19. I keep my feelings to myself *

*Mark only one oval.*

not at all agree

1

2

3

4

5

totally agree

20. In order not to feel bad about it (being sad/in a bad mood) I try to look at things from a different perspective *

*Mark only one oval.*

| | |
|---|---|
| | not at all agree |
| 1 | ◯ |
| 2 | ◯ |
| 3 | ◯ |
| 4 | ◯ |
| 5 | ◯ |
| | totally agree |

21. When I'm happy/happy, I try not to point it out *

*Mark only one oval.*

| | |
|---|---|
| | not at all agree |
| 1 | ◯ |
| 2 | ◯ |
| 3 | ◯ |
| 4 | ◯ |
| | ◯ |

22. When I have to face a difficult situation I try to look at it from a perspective that helps me stay calm *

*Mark only one oval.*

not at all agree

1 ⬭

2 ⬭

3 ⬭

4 ⬭

5 ⬭

totally agree

23. I control my emotions by not expressing them *

*Mark only one oval.*

not at all agree

1 ⬭

2 ⬭

3 ⬭

4 ⬭

⬭

24. Changing the way I think about a situation helps me feel better *

*Mark only one oval.*

| | not at all agree |
|---|---|
| 1 | ◯ |
| 2 | ◯ |
| 3 | ◯ |
| 4 | ◯ |
| 5 | ◯ |
| | totally agree |

25. I try to control my feelings by trying to change the way I look at the situation I'm in *

*Mark only one oval.*

| | not at all agree |
|---|---|
| 1 | ◯ |
| 2 | ◯ |
| 3 | ◯ |
| 4 | ◯ |
| | ◯ |

Machine Translated by Google


26. If I have negative feelings, I am careful not to express them *

*Mark only one oval.*

not at all agree

1

2

3

4

5

totally agree

27. Change the way you think about a situation , it helps me not feel bad *

*Mark only one oval.*

not at all agree

1

2

3

4

5

totally agree

*Go to question 30.*

Machine Translated by Google


## Quality of the couple relationship

In this section you will find questions aimed at a better understanding of the quality of the your relationship as a couple. If you are not currently in a relationship you can respond by doing reference to the last relationship had.

Machine Translated by Google


28. Romantic Relationship Quality *

*Mark only one oval per row.*

| | Absolutely false | Quite false | Neither true nor false | Enough True | Absolutely True |
|---|---|---|---|---|---|
| Me and my/my I pass all our partner pass all free time together | ⬭ | ⬭ | ⬭ | ⬭ | ⬭ |
| If I have any problem of study, work or study problem, at home i can talk to my partner about it | ⬭ | ⬭ | ⬭ | ⬭ | ⬭ |
| If others annoyed me my friend/partner my partner would help me would help me | ⬭ | ⬭ | ⬭ | ⬭ | ⬭ |
| My friend/ partner invents fun things to do to do | ⬭ | ⬭ | ⬭ | ⬭ | ⬭ |
| My friend/partner helps me when in trouble I'm in trouble | ⬭ | ⬭ | ⬭ | ⬭ | ⬭ |
| If my friend/ partner were to move to another city i think i would feel yours lack lack | ⬭ | ⬭ | ⬭ | ⬭ | ⬭ |
| When I do something well, something is good My friend/ partner is happy for me for me | ⬭ | ⬭ | ⬭ | ⬭ | ⬭ |

Machine Translated by Google


| | | | | | |
|---|---|---|---|---|---|
| Some time Some time My My friend / friend / partner / partner or things for me, what things for me, that make me feel special make me feel special special | | | | | |
| Some time Sometimes I I also quarrel quarrel very very violently violently with my friend with my friend/partner friend/partner | | | | | |
| If someone me If someone annoys me I think they annoy I think that my friend that my friend / friend / partner / partner / would defend would defend | | | | | |
| Some time Some time My My friend/ friend/ friend/ partner/ partner/ torments me and torments me and annoys me even if I annoy him even if I tell him not to [ I say not to [ | | | | | |
| If I had If I need money I need money I think I think My friend/ my friend/partner would give | | | | | |
| If after having If after arguing even arguing even violently violently with my/my/my/my friend/partner friend/partner I asked him/her I apologized I think I'm sorry I think he would continue to be ed would angry be angry with me with me | | | | | |
| Sometimes me Sometimes me and my as My friend/ friend/a/partners a/partners | | | | | |

Machine Translated by Google


together
and together
and we talk about
we talk about
study, work study
work and the things
we like and the
things we like

If I had
If I need I
need
something i
think something i
think my that my
friend/partner
friend/partner
would help me would
help me

If there is anything
If there is something
that
worries
me and worries me
and that I can't tell I
can't tell others I
tell the others I tell
my/my friend/my
friend/partner/partner

If I bother
If I annoy my friend /
my friend / and he /
she annoys and he /
she annoys me after
me after we make up
we make
up easily
easily

Some time
Some time
My
My friend/
partner friend/partner
we fight a lot we fight
a lot

Me and My
Me and My
friend/partner
friend/partner we
don't get
along we
don't agree
on we agree
on many
things many things

If I and My
If me and my
friend/partner
friend/partner
also argue we
also argue
violently we
apologize we
apologize we
apologize and
everything
returns to and
everything returns to its place

I'm happy
I am happy
when I am
when I am
together with
my friend
together with my
friend/partner friend/partner

I think about
my friend/partner

Machine Translated by Google


p even when he/she is not I think about my/ there friend/partner even when he/ she is not there

Machine Translated by Google


29. You will find a list of things that could happen when you have different perspectives with your partner. We ask you to tell us how many times this has happened in the last year. If they have not happened in the last year, but have previously, you can check the corresponding box (not last year but in the past). *

*Mark only one oval per row.*

| | it's never success | a once a year last | twice in the last year | 3-5 times in the last one year | 6-10 times in the last one year | 11 - 20 times in the last year | more than once in the last year |
|---|---|---|---|---|---|---|---|
| My partner has insulted. | ⬭ | ⬭ | ⬭ | ⬭ | ⬭ | ⬭ | ⬭ |
| threw something that could hurt me. | ⬭ | ⬭ | ⬭ | ⬭ | ⬭ | ⬭ | ⬭ |
| has twisted hair | ⬭ | ⬭ | ⬭ | ⬭ | ⬭ | ⬭ | ⬭ |
| I had one sprain, a bruise or a little cut because of a fight with my partner. | ⬭ | ⬭ | ⬭ | ⬭ | ⬭ | ⬭ | ⬭ |
| was sure we would be able to solve a problem | ⬭ | ⬭ | ⬭ | ⬭ | ⬭ | ⬭ | ⬭ |

Machine Translated by Google


he agreed to try a solution that i suggested
agreed to try a solution i have suggested

## Perceived stress

In this section you will find questions aimed at investigating the Stress you perceive.

30. Give an answer to each statement in the following questionnaire, referring to how you have felt in the last month. *

*Mark only one oval per row.*

| | Never | rarely | sometimes | often | always |
|---|---|---|---|---|---|
| **In the last** month, how often have you felt out of self since it happened **something unexpected?** | | | | | |
| **In the last** month, how often have you felt the feeling of not being able to have control over important things of his life? * | | | | | |
| **In the last month,** how often it was felt heard **nervous or "stressed"?** | | | | | |
| **In the last** month, how often have you felt confident about **Your ability** to manage your personal problems? * | | | | | |
| **In the last month,** how often did he have had the feeling that things were going he said | | | | | |

Machine Translated by Google


as he said as
eye said
She?
* She? *

In the last
In the last
month, with
what month, conche
frequency has
frequency has
had the
had the
feeling of feeling
of not being able
to not being
able to keep up
with keep up
with all"e
things c you"e
things c had to
do had to do

In the last
In the last
month, how
often, how often
have you
warned you
warned that
you are able
to control
what is
irritating
you what
is irritating you
in your life in
your life

In the last
In the last
month, how
often, how often
have you felt
like mastering
the
situation?
the situation?

In the last
In the last
month, how
often, how often
have you
been angry
about
things
that were out of
your control?

His control?

In the last
In the last
month, how
often, how often
have you had
the feeling that
the
difficulties
and
difficulties
were
piling
up to such
an extent
that he
could
not he could
not overcome
them?

overcome them?

## Work stress - related

In this section you will find questions aimed at investigating the management of work-related stress.

31. Please read the following statements about your work in the last 6 months carefully and indicate how often you experienced them using the scale below. *

*Mark only one oval per row.*

| | Never | Rarely | Some time | Often | Always |
|---|---|---|---|---|---|
| **I have to work very intensely** | | | | | |
| **I receive pressure to** after **work overtime** | | | | | |
| I have freedom of choice deciding **what to do at work** | | | | | |
| I have to do my job very much quickly quickly | | | | | |
| **At work are subject to bullying and harassment** | | | | | |
| I have deadlines impossible time impossible to meet to meet | | | | | |

Machine Translated by Google


Sensory self-assessment

The following questions investigate sensory and physiological changes.

32. Please indicate the answer that describes you as accurately as possible. *

*Mark only one oval per row.*

| | Never | rarely | sometimes | often | always |
|---|---|---|---|---|---|
| **Mouth** dry dry | | | | | |
| Breath quick / Quick breath | | | | | |
| Vulsgular tension in the arms and in the legs *legs* | | | | | |
| Sensation of swelling due to retention water * | | | | | |
| **Goose bumps'** | | | | | |
| **Ache stomach** | | | | | |
| Tension or **swelling** in the ear **stomach** | | | | | |
| **Tremor** of the lips *lips* | | | | | |
| **Sensation to have** i spiky hair **neck** | | | | | |
| **Need to swallow** | | | | | |
| **Beat** rapid heart heart rate | | | | | |

Machine Translated by Google


33. Read each sentence carefully and indicate if and, if any, how much discomfort each symptom has caused you in the past week (including today). *

*Mark only one oval per row.*

| | for nothing | a little | quite | a lot |
|---|---|---|---|---|
| Numbness or pins and needles | ⬭ | ⬭ | ⬭ | ⬭ |
| Hot flashes of heat | ⬭ | ⬭ | ⬭ | ⬭ |
| Wobbly legs | ⬭ | ⬭ | ⬭ | ⬭ |
| Inability to relax | ⬭ | ⬭ | ⬭ | ⬭ |
| Afraid that something bad may happen | ⬭ | ⬭ | ⬭ | ⬭ |
| dizziness or feeling lightheaded daze | ⬭ | ⬭ | ⬭ | ⬭ |
| heartbeat | ⬭ | ⬭ | ⬭ | ⬭ |
| mood unstable | ⬭ | ⬭ | ⬭ | ⬭ |
| To be terrified | ⬭ | ⬭ | ⬭ | ⬭ |
| Feeling agitated | ⬭ | ⬭ | ⬭ | ⬭ |
| Feeling of suffocation | ⬭ | ⬭ | ⬭ | ⬭ |
| Hands that tremble | ⬭ | ⬭ | ⬭ | ⬭ |
| Shaking in the whole body | ⬭ | ⬭ | ⬭ | ⬭ |
| Fear of losing control | ⬭ | ⬭ | ⬭ | ⬭ |

| | | | | |
|---|---|---|---|---|
| Breath Heavy wheezing | | | | |
| Fear of Fear of dying dying | | | | |
| Feel Feeling afraid afraid | | | | |

## Pains

| | | | | |
|---|---|---|---|---|
| Intestinal or intestinal or stomach pains | | | | |
| Feel Feeling faint | | | | |
| Feel Feeling blushing blushing | | | | |
| Feeling sweaty Feeling sweaty (not because (not because of the heat) of the heat) | | | | |

34. How would you rate your headache on a scale of 0 to 10 right now (right now)? *

*Mark only one oval.*

no pain

0 ◯
1 ◯
2 ◯
3 ◯
4 ◯
5 ◯
6 ◯
7 ◯
8 ◯
9 ◯
10 ◯

maximum pain

35. In the past 3 months, how intense was your worst pain? *

*Mark only one oval.*

| | no pain |
|---|---|
| 0 | ⬭ |
| 1 | ⬭ |
| 2 | ⬭ |
| 3 | ⬭ |
| 4 | ⬭ |
| 5 | ⬭ |
| 6 | ⬭ |
| 7 | ⬭ |
| 8 | ⬭ |
| 9 | ⬭ |
| 10 | ⬭ |
| | maximum pain |

36. In the last 3months on average how intense was your headache? *

*Mark only one oval.*

no pain

0 ⬭

1 ⬭

2 ⬭

3 ⬭

4 ⬭

5 ⬭

6 ⬭

7 ⬭

8 ⬭

9 ⬭

10 ⬭

maximum pain

Machine Translated by Google


37. How much has your headache interfered with your daily activities in the past 3 months? *

*Mark only one oval.*

for nothing

0 ◯

1 ◯

2 ◯

3 ◯

4 ◯

5 ◯

6 ◯

7 ◯

8 ◯

9 ◯

10 ◯

very very much

38. How much has your headache affected your ability to participate in family social recreational activities in the past 3 months? *

*Mark only one oval.*

no changes

0 ◯
1 ◯
2 ◯
3 ◯
4 ◯
5 ◯
6 ◯
7 ◯
8 ◯
9 ◯
10 ◯

extreme change

39. How much has your headache changed your ability to do work (including housework) in the last 3 months? *

*Mark only one oval.*

| | no changes |
|---|---|
| 0 | ⬭ |
| 1 | ⬭ |
| 2 | ⬭ |
| 3 | ⬭ |
| 4 | ⬭ |
| 5 | ⬭ |
| 6 | ⬭ |
| 7 | ⬭ |
| 8 | ⬭ |
| 9 | ⬭ |
| 10 | ⬭ |
| | extreme change |

Machine Translated by Google


40. On how many days in the last 3 months have you been kept away from your usual activities because of your Headache (compute on average in 1 month) *

*Mark only one oval.*

- 0 - 6 days
- 7 - 14 days
- 15 - 30 days
-
-

This content is neither created nor endorsed by Google.

Google Forms
