# Supplementary material for: Gender-related stress factors and emotional perception in migraine: a structured online questionnaire in migraine patients and controls
Source: Neurol Sci. 2023 Nov 7;45(4):1645–54. doi: 10.1007/s10072-023-07152-6 (PMC10942877; doi:10.1007/s10072-023-07152-6)
Supplement: Supplementary file 2 — Supplementary file2 (PDF 1754 KB) [file 10072_2023_7152_MOESM2_ESM.pdf]

Influenze di genere nel dolore cronico l'emicrania

Gentile Signora/e,

stiamo conducendo una ricerca sull'influenza dello stress lavorativo e familiare sulla sua patologia dolorosa cronica . Questo studio è coordinato dall'Università di Bari, AOU Policlinico.
Responsabile scienti co dello studio è la prof.ssa Marina de Tommaso

La partecipazione consiste nella compilazione di alcuni questionari, vi ruberà una decina di minuti del vostro tempo.

La preghiamo di leggere attentamente quanto riportato di seguito.
L'Azienda Ospedaliero Universitaria Policlinico di Bari quali Titolare del trattamento dei dati, e il responsabile interno del trattamento, tratteranno i suoi dati personali, in particolare quelli riguardanti la salute e, soltanto nella misura in cui siano indispensabili in relazione all'obiettivo dello studio, altri dati riguardanti la sua origine, e i dati genetici, esclusivamente in funzione della realizzazione dello studio.
La persona autorizzata al trattamento dati sotto l'autorità del Titolare è la Prof.ssa Marina de Tommaso presso la Clinica Neurologica Amaducci/Neuro siopatologia

1. FINALITÀ DEL TRATTAMENTO

Ricerca scienti ca e statistica nalizzata alla tutela della salute della collettività in campo medico, biomedico ed epidemiologico.

2. NATURA DEI DATI (quali dati verranno raccolti e trattati) I dati raccolti e trattati saranno dati:

- dati personali : qualsiasi informazione riguardante una persona sica, compreso un numero di identi cazione a prescindere dal supporto utilizzato (cartaceo, elettronico, ecc);
- dati sensibili, ad esempio quelli riguardanti lo stato di salute e l'origine

3. BASE GIURIDICA DEL TRATTAMENTO

I dati personali che fornirà per le nalità di cui al punto 1 verranno trattati esclusivamente sulla base del Suo espresso consenso, che potrà revocare in ogni momento. L'eventuale revoca avrà valore solo per il futuro, restando valido il trattamento eseguito no a quel momento. I dati raccolti no al momento della revoca verranno inclusi nello studio, i successivi eliminati.

4. NATURA E CONSEGUENZE DEL CONFERIMENTO DEI DATI

Il conferimento dei Suoi dati, assolutamente facoltativo, è però necessario per la partecipazione allo studio o, comunque, per il perseguimento delle nalità sopra richiamate. Il mancato conferimento dei dati può impedire, in tutto o in parte, la partecipazione allo studio. La partecipazione allo studio avviene su base volontaria, pertanto, il mancato consenso al trattamento dei dati per tale nalità non pregiudica il diritto di avvalersi delle altre prestazioni medico-sanitarie erogate dal Centro.

5. MODALITÀ DI TRATTAMENTO DEI DATI

Le operazioni di raccolta, registrazione, conservazione e modi cazione dei dati personali avverranno mediante strumenti informatici con logiche strettamente correlate alle nalità di cui al punto 1. I dati verranno trattati applicando al trattamento le misure adeguate di sicurezza ai sensi degli artt. 32 e seguenti del Regolamento UE 2016/679 e, ciò, sia sotto un pro lo organizzativo sia sotto un pro lo tecnologico. I suoi dati personali verranno raccolti con l'utilizzo di un codice che non consentirà la Sua diretta identi cazione, perché la documentazione che consente di recuperare

la Sua identità verrà conservata e custodita dallo Sperimentatore, separatamente dai documenti, e sarà accessibile solo a soggetti speci camente da lui autorizzati.

6. COMUNICAZIONE E DIFFUSIONE
I suoi dati personali non potranno essere diffusi, se non in forma aggregata e, quindi, in modo assolutamente anonimo. I Suoi dati potranno essere resi disponibili a soggetti, anche esterni, espressamente designati quali Responsabili del trattamento, in modalità elettronica pseudoanonimizzata. (vale a dire non associati al suo nome, ma a un codice). 7. DURATA DEL TRATTAMENTO
I dati da Lei forniti e successivamente elaborati saranno conservati per un arco di tempo non superiore a quello necessario per conseguire le nalità per le quali sono stati raccolti e trattati e, quindi, per la durata di 36 mesi.
8. ESERCIZIO DEI DIRITTI
Lei potrà esercitare in qualsiasi momento i diritti dell'interessato, come disciplinati dagli artt. 13 e seguenti del Regolamento UE 2016/679. In ogni caso lei potrà in qualsiasi momento, e nei casi previsti dalla legge, chiedere l'accesso, la retti ca e la cancellazione dei dati, la loro portabilità presso altro titolare, così come potrà chiedere la limitazione del trattamento ed opporsi allo stesso. Potrà inoltre revocare il consenso, potendo così interrompere in ogni momento e senza fornire alcuna giusti cazione la Sua partecipazione allo studio; in tal caso non saranno più raccolti ulteriori dati che la riguardano, ferma restando l'utilizzazione di quelli eventualmente già raccolti secondo quanto indicato al punto 3. Per l'esercizio di tali diritti lei può presentare richiesta, attraverso la modalità che ritiene più opportuna, direttamente al titolare del trattamento, al responsabile o anche al personale. Può inoltre scrivere ai Responsabili della Protezione dei dati designati dal titolare stesso, contattabili attraverso i seguenti indirizzi mail: marina.detommaso@uniba.it; gianni.lucatorto@policlinico.ba.it

Lei gode altresì del diritto di presentare reclamo all'autorità di controllo anche del suo paese di residenza. Nel caso in cui sia residente in Italia l'autorità alla quale presentare il reclamo è il Garante per la protezione dei dati, per le cui istruzioni può collegarsi al link https://www.garanteprivacy.it/web/guest/home/docweb/-/docwebdisplay/docweb/4535524

8. Cookies tecnici
Nessun dato personale degli utenti viene in proposito acquisito dal sito. Non viene fatto uso di cookies per le informazioni di carattere personale, né vengono utilizzati cookies persistenti di alcun tipo ovvero sistemi per il tracciamento utenti. L'uso dei cookies di sessione (che non vengono memorizzati in modo persistente sul computer dell'utente e svaniscono con la chiusura del browser) è strettamente limitato alla trasmissione di identi cativi di sessione necessari per consentire l'esplorazione sicura ed e ciente del sito. I cookies di sessione utilizzati in questo sito evitano il ricorso ad altre tecniche informatiche potenzialmente pregiudizievoli per la riservatezza della navigazione degli utenti e non consentono l'acquisizione di dati personali identi cativi dell'utente.

9. Cookie di terze parti
Non si utilizzeranno inoltre cookie provenienti da terze parti.

10. Per quali nalità trattiamo i Suoi dati?
Il trattamento dei suoi dati personali e "particolari" (quali ad esempio quelli idonei a rivelare lo stato di salute e/o la vita sessuale della persona, l'origine razziale o etnica, le convinzioni religiose ed i dati genetici) sarà effettuato esclusivamente per le nalità di seguito esposte:

10.1. nalità di studio e ricerca

11. Basi giuridiche che legittimano il trattamento
Il trattamento dei dati raccolti è legittimato dagli art. del GDPR, come esplicitato di seguito per ogni speci ca nalità, di cui ai seguenti punti della presente informativa:

12. A chi comunichiamo i Suoi dati?
I dati non saranno comunicati ad alcuno

I dati oggetto del trattamento, in quanto idonei a rivelare lo stato di salute non saranno diffusi.

13. Modalità di erogazione dei servizil servizi sono erogati mediante l'utilizzo di: WEBSurvey;

14. Per quanto tempo conserviamo i Suoi dati?
Tutti i dati personali acquisiti attraverso il Google Moduli saranno trattati n quando perdura il periodo di valutazione. Concluso tale periodo, i dati saranno conservati per 01 anno e successivamente cancellati.

15. Quali obblighi abbiamo nei Suoi confronti?
Abbiamo l'obbligo di rispondere alle Sue richieste e, speci camente di consentirLe di esercitare i Suoi diritti come di seguito esplicitato:

a) Accesso: conoscere quali dati trattiamo, come e perché li trattiamo
b) Retti ca: correggere i dati personali inesatti, ove applicabile
c) Cancellazione (diritto all'oblio)
d) Limitazione del trattamento: attuabile solo per alcune speci che situazioni
e) Ricevere comunicazione in caso di retti ca, cancellazione, limitazione
f) Diritto alla portabilità dei dati: non applicabile per i trattamenti effettuati sulla base di una norma di legge o di regolamento e per i dati "derivati" quali ad esempio le valutazioni riguardanti lo stato di salute (referti e documentazione sanitaria in genere)
g) Diritto di opposizione: l'interessato ha il diritto di opporsi in qualunque momento al trattamento per nalità ulteriori a quelle direttamente o indirettamente connesse al ricovero per ni di diagnosi, assistenza e terapia sanitaria, fatta salva l'esistenza di motivi legittimi che prevalgono sugli interessi, sui diritti e sulle libertà fondamentali dell'interessato oppure per l'accertamento, l'esercizio o la difesa di un diritto in sede giudiziaria
h) Diritto di revoca del consenso: applicabile esclusivamente ai trattamenti effettuati sulla base del rilascio del consenso rimanendo tuttavia valido per i trattamenti effettuati precedentemente alla revoca
i) Diritto di proporre un reclamo ad un'Autorità di Controllo: nel caso in cui ritenesse di non avere ricevuto risposte adeguate alle Sue richieste potrà rivolgersi all'Autorità Garante Privacy dello stato in cui risiede o lavora o proporre un ricorso dinanzi all'autorità giudiziaria

16. È obbligato a fornire i Suoi dati?
A parte quanto speci cato per i dati di navigazione, l'utente è libero di fornire i dati personali o comunque indicati in contatti telefonici e nel corso della gestione clinica . Il loro mancato conferimento può comportare l'impossibilità di ottenere quanto richiesto Una volta che l'utente abbia deciso di utilizzare i servizi offerti dalla WEBAPP, il conferimento dei dati è necessario per la corretta erogazione dei servizi stessi e la mancata trasmissione degli stessi o una loro parziale o inesatta trasmissione comporterà l'impossibilità per l'utente di fruire tali servizi. Il trattamento dei dati suindicati è effettuato per le nalità di cui sopra, dai sistemi Informativi con i quali la WEBAPP coopera, in conformità a quanto stabilito all'articolo 12 del RGDP per mezzo di strumenti elettronici o comunque automatizzati, nel rispetto delle regole di riservatezza e di sicurezza previste dalla legge e/o dalla normativa regolamentare.

17. Da dove hanno origine i Suoi dati?
I dati personali necessari per la corretta attuazione delle attività direttamente o indirettamente connesse alle prestazioni sanitarie erogate in regime ambulatoriale sono forniti direttamente dall'interessato.

18. Reclamo all'autorità di controllo.
Ai sensi dell'art. 77 del Regolamento UE 2016/679, Le ricordiamo che Lei ha il diritto di proporre reclamo all'Autorità di Controllo (Garante per la Protezione dei dati personali), nel caso in cui ritenga che il trattamento che la riguarda violi le disposizioni del Regolamento medesimo.

19. Esistenza di processi decisionali automatizzati nel trattamento.
Si speci ca che per il trattamento dei dati di cui sopra NON è presente nessun tipo di processo decisionale automatizzato, ai sensi dell'art. 22 del Regolamento UE 2016/679
20. Non è previsto alcun trasferimento di dati personali al di fuori dell'U.E.

A chi rivolgersi e come esercitare i Suoi diritti?
Per far valere i suoi diritti come previsto al CAPO III del Regolamento 2016/679 (sinteticamente esplicitati al punto 7) può rivolgersi al Responsabile della protezione dei dati personali dell' AOU Policlinico di Bari ,

Responsabile del progetto di ricerca alla quale chiedere ulteriori ed eventuali chiarimenti:

Prof.ssa Marina de Tommaso marina.detommaso@uniba.it

Se in accordo con quanto su riportato, può cliccare su SI

~~* Indica una domanda obbligatoria~~

Titolo predefinito

Sezione senza titolo

Sezione senza titolo

1.

Seleziona tutte le voci applicabili.

☐ SI

Caratteristiche anagrafiche

In questa sezione troverai domande volte a raccogliere alcune informazioni di carattere anagra co e connessi alla tua storia.
Come hai letto nella sezione precedente tutti i dati saranno trattati in modo anonimo.

2. Genere *

Contrassegna solo un ovale.

- Uomo
- Donna
- Altro

3. Quanti anni hai *

Contrassegna solo un ovale.

- tra i 20 e i 30
- tra i 31 e i 40
- tra i 41 e i 50
- tra i 51 e i 60
- tra i 61 e i 70
- tra i 71 e gli 80
- 4.

5. Titolo di studio *

Contrassegna solo un ovale.

- diploma sc superiore
- laurea formazione post
- laurea nessun titolo
-
-
- 6. Stato civile *

Contrassegna solo un ovale.

- Nubile
- Coniugata Separata
- Vedova
-

7. In quale regione vivi? *

Contrassegna solo un ovale.

- Abruzzo
- Basilicata
- Calabria
- Campania
- Emilia Romagna
- Friuli Venezia Giulia
- Lazio
- Liguria
- Lombardia
- Marche
- Molise
- Piemonte Puglia
- Sardegna
- Sicilia
- Toscana
- Trentino Alto Adige Umbria
- Val d'Aosta
- Veneto

7. Situazione lavorativa attuale * *Contrassegna solo un ovale.*

- disoccupata/o occupazione saltuaria (part- time/ a
- chiamata) occupazione regolare (full - time /
- tempo pieno)

8. Sei una fumatrice/fumatore? *

Contrassegna solo un ovale.

- SI
- NO

9. Pensi di essere stata/o mai oggetto di uno o più dei seguenti tipi di violenza ? (* può indicare più risposte)

Contrassegna solo un ovale.

- Fisica
- Psicologica
- Economica
- Sessuale
- Stalking
- Mobbing Minacce
- nessuna delle precedenti
- Altro:
- __________________

10. Attualmente hai una relazione di coppia? Se risponde no puo' saltare la sezione 4 *

Contrassegna solo un ovale.

- Si
- No

11. Da quanto tempo hai una relazione di coppia ?

Contrassegna solo un ovale.

- Meno di un anno
- Da uno a tre anni
- Più di tre anni

12. Hai figli? *

Contrassegna solo un ovale.

- Si minorenni
- Si maggiorenni
- Si, sia minorenni che maggiorenni
- NO

13. I tuoi figli vivono con te? *

Contrassegna solo un ovale.

- Si
- No
- Non ho gli

14. Hai, oltre i figli, altri familiari di cui ti prendi cura? *

Contrassegna solo un ovale.

- Si
- No

15. Se hai risposto si alla precedente domanda, puoi indicare se sono :

Contrassegna solo un ovale.

- Anziani
- Persone con disabilità siche
- Persone con disabilità cognitive
- Minori
- Nessuna delle Precedenti Altro:
- ______________________

Questionario sulla regolazione emotiva (ERQ)

Qui di seguito, ti chiediamo di rispondere ad alcune domande sulla tua esperienza emotiva, in particolare riguardo al modo in cui controlli (cioè regoli e gestisci) le tue emozioni. Le domande comprendono due diversi aspetti circa le emozioni. Il primo aspetto riguarda la tua esperienza, ovvero quello che provi, senti dentro. Il secondo riguarda invece l'espressione, cioè il modo in cui mostri le tue emozioni nel modo di parlare, esprimerti e comportarti. Nonostante alcune domande ti sembreranno simili, in realtà essere differiscono per alcuni aspetti importanti. Ti chiediamo quindi di leggere con attenzione e di rispondere utilizzando questa scala di valori.

*

18. Per sentirmi meglio (felice/contento/sollevato/di buon umore) cerco di guardare le cose da una prospettiva diversa *Contrassegna solo un ovale.*

	Per niente d'accordo
1	◯
2	◯
3	◯
4	◯
5	◯
	Totalmente d'accordo

	per niente d'accordo
1	◯
2	◯
3	◯
4	◯
5	
	totalmente d'accordo

*

19. Tengo i miei sentimenti per me *

Contrassegna solo un ovale.

20. Per non starci male (essere triste/ di cattivo umore) cerco di guardare le cose da una prospettiva diversa *Contrassegna solo un ovale.*

per niente d'accordo

5

totalmente d'accordo

*

1

2

3

4

5

totalmente d'accordo

21. Quando sono contento/felice, cerco di non farlo notare *

Contrassegna solo un ovale.

22. Quando devo affrontare una situazione difficile cerco di considerarla da una prosettiva che mi aiuti a stare calma *Contrassegna solo un ovale.*

per niente d'accordo

per niente d'accordo

1

2

3

4

5

totalmente d'accordo

*

1 ⬭
2 ⬭
3 ⬭
4 ⬭
5 ⬭

totalmente d'accordo

23. Controllo le mie emozioni non esprimendole *

Contrassegna solo un ovale.

per niente d'accordo

1 ⬭
2 ⬭
3 ⬭
4 ⬭
5

totalmente d'accordo

24. Cambiare il modo di pensare ad una situazione mi aiuta a sentirmi meglio *

Contrassegna solo un ovale.

per niente d'accordo	
1	◯
2	◯
3	◯
4	◯
5	◯
totalmente d'accordo	

25. Cerco di controllare i miei sentimenti provando a cambiare il modo di considerare la situazione in cui mi trovo *

Contrassegna solo un ovale.

per niente d'accordo	
1	◯
2	◯
3	◯
4	◯
5	◯
totalmente d'accordo	

26. Se provo sentimenti negativi faccio attenzione a non esprimerli *

Contrassegna solo un ovale.

	per niente d'accordo
1	⬭
2	⬭
3	⬭
4	⬭
5	⬭
	totalmente d'accordo

27. Cambiare il modo di pensare ad una situazione , mi aiuta a non starci male *

Contrassegna solo un ovale.

	per niente d'accordo
1	⬭
2	⬭
3	⬭
4	⬭
5	⬭
	totalmente d'accordo

Passa alla domanda 30.

Qualità della relazione di coppia

In questa sezione troverai domande volte ad una maggiore comprensione della qualità del tuo rapporto di coppia. Se attualmente non hai una relazione puoi rispondere facendo riferimento all'ultima relazione avuta.

28. Romantic Relationship Quality *

Contrassegna solo un ovale per riga.

	Assolutamente falso	Abbastanza falso	Né vero né falso	Abbastanza vero	Assolutamente vero
Io e il/la mio/ mia partner passiamo tutto il nostro tempo libero insieme					
Se ho qualche problema di studio, di lavoro o a casa posso parlarne con il/la mio/mia partner					
Se gli altri mi dessero noia il/la mio/a amico/a partner mi aiuterebbe					
Il/la mio/a amico/ a/partner inventa delle cose divertenti da fare					
Il/La mio amico/a/partner mi aiuta quando sono in di coltà					
Se Il/la mio/a amico/ a/partnerdovesse trasferirsi in un'altra città penso che sentirei la sua mancanza					
Quando faccio bene qualcosa Il/la mio/a amico/ a/partnerè contento per me					
Qualche voltaQualche volta Il/la mio/aIl/la mio/a amico/amico/ a/partnerfa dellea/partnerfa delle cose per me, checose per me, che mi fanno sentiremi fanno sentire specialespeciale					
Qualche voltaQualche volta litigo anchelitigo anche moltomolto violentementeviolentemente con Il/la mio/acon Il/la mio/a amico/ a/partneramico/ a/partner					
Se qualcuno miSe qualcuno mi desse noia pensodesse noia penso che Il/la mio/ache Il/la mio/a amico/amico/ a/partnermia/partnermi difenderebbedifenderebbe					

Qualche voltaQualche volta Il/la mio/aIl/la mio/a amico/amico/ a/partnermia/partnermi tormenta e mi datormenta e mi da noia anche se glinoia anche se gli dico di non farlo [dico di non farlo [

Se avessiSe avessi bisogno di soldibisogno di soldi penso che Il/lapenso che Il/la mio/a amico/mio/a amico/ a/partnerme lia/partnerme li darebbedarebbe

Se dopo averSe dopo aver litigato anchelitigato anche violentementeviolentemente con gIl/la mio/acon gIl/la mio/a amico/ a/partneramico/ a/partner gli/le chiedessigli/le chiedessi scusa penso chescusa penso che egliegli continuerebbe adcontinuerebbe ad essere arrabbiatoessere arrabbiato con mecon me

Qualche volta ioQualche volta io e Il/la mio/ae Il/la mio/a amico/amico/ a/partnerstiamoa/partnerstiamo
i i
insieme einsieme e
parliamo
delloparliamo dello
studio, del
lavorostudio, del
lavoro e delle cose
chee delle cose che
ci piaccionoci
piacciono

Se avessiSe avessi bisogno dibisogno di qualcosa pensoqualcosa penso che Il/la mio/ache Il/la mio/a amico/ a/partneramico/ a/partner mi aiuterebbemi aiuterebbe

Se c'è qualcosaSe c'è qualcosa che miche mi preoccupa e chepreoccupa e che non posso direnon posso dire
ad altri la dico alad altri
la dico al mio/a
amico/mio/a amico/
a/partnera/partner

Se io do fastidioSe io do fastidio al Imio/a amico/al Imio/a amico/ e lui/lei da noia ae lui/lei da noia a
me dopome dopo facciamo la pacefacciamo la pace

facilmente
facilmente

Qualche volta Il/la mio/a amico/ a/partner litighiamo molto
Qualche volta Il/la mio/a amico/ a/partner litighiamo molto

Io e Il/la mio/a
Io e Il/la mio/a
amico/ a/partneramico/ a/partner non andiamonon andiamo d’accordo sud’accordo su molte cosemolte cose

Se io e Il/la mio/aSe io e Il/la mio/a amico/ a/partneramico/ a/partner litighiamo anchelitighiamo anche violentemente civiolentemente ci chiediamo scusachiediamo scusa e tutto torna ae tutto torna a posto
posto

Sono contento quando sono insieme al mio/a amico/ a/partner
Sono contento quando sono insieme al mio/a amico/ a/partner

Penso al mio/a amico/ a/partner p anche quando Penso al

mio/a lui/lei non c’è amico/ a/partner anche quando lui/lei non c’è

29. Troverai un elenco di cose che potrebbero accadere quando si hanno delle prospettive differenti con il/la proprio/a partner. Ti chiediamo di indicarci quante volte sono accadute nell'ultimo anno. Se non sono accadute nel corso dell'ultimo anno, ma in precedenza si, puoi selezionare la casella corrispondente (non l'anno scorso ma in passato). *

Contrassegna solo un ovale per riga.

	non è mai successo	una volta l'anno scorso	due volte nell'ultimo anno	3-5 volte nell'ultimo anno	6-10 volte nell'ultimo anno	11 - 20 volte nell'ultimo anno	più di volte nell'ulti anno
Il/la mio/a partner mi ha insultato.							
mi ha lanciato qualcosa che avrebbe potuto ferirmi.							
mi ha storto il braccio o i capelli							
Ho avuto una distorsione, un livido o un piccolo taglio a causa di una lite con il mio partner.							
era sicuro/a che avremmo potuto risolvere un problema							
ha ha accettato accettato di provare di provare una una soluzione soluzione che ho che ho suggerito suggerito							

Stress Percepito

In questa sezione troverai domande volte a indagare lo Stress da te percepito.

30. Dai una risposta ad ogni affermazione del seguente questionario, in riferimento * a come ti sei sentita nell'ultimo mese.

Contrassegna solo un ovale per riga.

	Mai	raramente	a volte	spesso	sempre
Nell’ultimo mese, con che frequenza si è sentita fuori di sé poiché è avvenuto qualcosa di inaspettato?					
Nell’ultimo mese, con che frequenza ha avuto la sensazione di non essere in grado di avere controllo sul cose importanti della Sua vita? *					
Nell’ultimo mese, con che frequenza si è sentito nervosa o “stressata”?					
Nell’ultimo mese, con che frequenza si è sentito duciosa sulla Sua capacità di gestire i suoi problemi personali? *					
Nell’ultimo mese, con che frequenza ha avuto la sensazione che le cose andassero come diceva come diceva Lei? *come diceva Lei? *					
Nell’ultimo mese, con che Nell’ultimo frequenza hamese, con che avuto lafrequenza ha sensazione di avuto la non riuscire a sensazione di star dietro anon riuscire a tu"e le cose c star dietro a doveva faretu"e le cose c doveva fare					
Nell’ultimo mese, con che Nell’ultimo frequenza hamese, con che avvertito difrequenza ha essere inavvertito di grado diessere in controllare ciò grado di che la irritacontrollare ciò nella Sua vita che la irrita nella Sua vita					

Nell'ultimo

mese, con che Nell'ultimo frequenza hamese, con che sentito difrequenza

ha padroneggiare sentito

di la situazione? padroneggiare la situazione?

Nell'ultimo

mese, con che Nell'ultimo frequenza èmese, con che statofrequenza è

arrabbiato per stato cose

chearrabbiato per erano fuori dal cose che

Suo controllo?erano fuori dal

Suo controllo?

Nell'ultimo

mese, con che Nell'ultimo frequenza hamese, con che avuto lafrequenza

ha sensazione avuto la che lesensazione di che le coltà si stavanodi coltà si

accumulando sta

vano a punto

tale accumuland

o

per cui nona punto

tale potevaper cui

non

superarle? poteva

superarle?

Stress lavoro - correlato

In questa sezione troverai domande volte ad indagare la gestione dello stress lavoro - correlato.

31. Per favore legga con attenzione le seguenti affermazioni relative al Suo lavoro negli ultimi 6 mesi ed indichi quanto frequentemente le ha vissute utilizzando la scala sotto riportata. *

Contrassegna solo un ovale per riga.

	Mai	Raramente	Qualche volta	Spesso	Sempre
Devo lavorare molto intensamente					

Ricevo pressioni per lavorare oltre l'orario	⬭	⬭	⬭	⬭	⬭
Ho libertà di scelta nel decidere cosa fare al lavoro	⬭	⬭	⬭	⬭	⬭
Devo svolgere il mio lavoro molto velocemente	⬭	⬭	⬭	⬭	⬭
Al lavoro sono soggetto a prepotenze e vessazioni	⬭	⬭	⬭	⬭	⬭
Ho scadenze temporali impossibili da rispettare	⬭	⬭	⬭	⬭	⬭

Autovalutazione sensoriale

Le seguenti domande indagano modi che sensoriali e siologiche.

32. Indica la risposta che ti descrive quanto più accuratamente possibile. *

Contrassegna solo un ovale per riga.

	Mai	raramente	a volte	spesso	sempre
Bocca asciutta	⬭	⬭	⬭	⬭	⬭
Respiro veloce	⬭	⬭	⬭	⬭	⬭
Tensione muscolare nelle braccia e nelle gambe *	⬭	⬭	⬭	⬭	⬭
Sensazione di gon ore dovuto a ritenzione idrica *	⬭ ⬭ ⬭			⬭	⬭
Pelle d'oca'	⬭	⬭	⬭	⬭	⬭
Mal di stomaco	⬭	⬭	⬭	⬭	⬭
Tensione o gon ore allo stomaco	⬭	⬭	⬭	⬭	⬭
Tremore delle labbra *	⬭	⬭	⬭	⬭	⬭
Sensazione di avere i peli irti sul	⬭	⬭	⬭	⬭	⬭
Bisogno di deglutire	⬭	⬭	⬭	⬭	⬭
Battito cardiaco accelerato	⬭	⬭	⬭	⬭	⬭

collo

33. Leggi attentamente ogni frase e indichi se ed, eventualmente, quanto fastidio * ciascun sintomo ti ha causato nella scorsa settimana (incluso oggi).

Contrassegna solo un ovale per riga.

	per niente	un pò	abbastanza	molto
Intorpidimento o formicolio	⬭	⬭	⬭	⬭
Vampate di calore	⬭	⬭	⬭	⬭
Gambe vacillanti	⬭	⬭	⬭	⬭
Incapacità a rilassarsi	⬭	⬭	⬭	⬭
Paura che qualcosa di molto brutto possa accadere	⬭	⬭	⬭	⬭
vertigini o sensazione di stordimento	⬭	⬭	⬭	⬭
batticuore	⬭	⬭	⬭	⬭
umore instabile	⬭	⬭	⬭	⬭
Essere terrorizzati	⬭	⬭	⬭	⬭
Sentirsi agitati	⬭	⬭	⬭	⬭
Sensazione di soffocamento	⬭	⬭	⬭	⬭
Mani che tremano	⬭	⬭	⬭	⬭
Agitazione in tutto il corpo	⬭	⬭	⬭	⬭
Paura di perdere il controllo	⬭	⬭	⬭	⬭

Respiro affannoso				
Paura di morire				
Sentirsi impauriti				
Dolori intestinali o di stomaco				
Sentirsi svenire				
Sentirsi arrossire				
Sentirsi sudati (non a causa del caldo)				

34. Come valuteresti la tua cefalea su una scala da 0 a 10 in questo momento (proprio ora)? *

Contrassegna solo un ovale.

	nessun dolore
0	◯
1	◯
2	◯
3	◯
4	◯
5	◯
6	◯
7	◯
8	◯
9	◯
10	◯
	massimo dolore

35. Negli ultimi 3 mesi quanto è stata intenso il tuo peggior dolore? *

Contrassegna solo un ovale.

	nessun dolore
0	⬭
1	⬭
2	⬭
3	⬭
4	⬭
5	⬭
6	⬭
7	⬭
8	⬭
9	⬭
10	⬭
	massimo dolore

36. Negli ultimi 3 mesi in media quanto è stata intensa la tua cefalea? *

Contrassegna solo un ovale.

nessun dolore	
0	◯
1	◯
2	◯
3	◯
4	◯
5	◯
6	◯
7	◯
8	◯
9	◯
10	◯
massimo dolore	

37. Negli ultimi 3 mesi quanto ha interferito la cefalea con le tue attività quotidiane? *

Contrassegna solo un ovale.

*

	per niente
0	⬭
1	⬭
2	⬭
3	⬭
4	⬭
5	⬭
6	⬭
7	⬭
8	⬭
9	⬭
10	⬭
	moltissimo

38. Negli ultimi 3 mesi quanto la tua cefalea ha cambiato la tua capacità di prendere parte alle attività ricreative sociali familiari?

Contrassegna solo un ovale.

*

nessuna modica

0

1

2

3

4

5

6

7

8

9

10

cambiamento estremo

39. Negli ultimi 3 mesi quanto la tua cefalea ha cambiato la tua capacità di lavorare (inclusi i lavori di casa)? *

Contrassegna solo un ovale.

*

nessuna modi ca

0

1

2

3

4

5

6

7

8

9

10

cambiamento estremo

40. Su quanti giorni negli ultimi 3 mesi sei stata/o tenuta/o lontano dalle tue solite attività a causa della tua cefalea (mese tipo valutato negli ultimi 3 mesi)?

*

Contrassegna solo un ovale.

- 0 - 6 giorni
- 7 - 14 giorni
- 15 - 30 giorni

Questi contenuti non sono creati né avallati da Google.

Google Moduli
