# Supplementary material for: Gender-related stress factors and emotional perception in migraine: a structured online questionnaire in migraine patients and controls
Source: Neurol Sci. 2023 Nov 7;45(4):1645–54. doi: 10.1007/s10072-023-07152-6 (PMC10942877; doi:10.1007/s10072-023-07152-6)
Supplement: Supplementary file 3 — Supplementary file3 (DOCX 12 KB) [file 10072_2023_7152_MOESM3_ESM.docx]

Supplementary material

METHODS

Perceptive functioning

Body perception

The Body Perception Questionnaire (BPQ-VSF) is a self-report measure of body awareness. A shorter version of the 12-item body awareness subscale that investigated body awareness. Each item was asked to rate it on a Likert scale from 1 to 5, with 1 indicating "Never", 2 "Occasionally", 3 "Sometimes", 4 "Usually" and 5 "Always".

Perceived stress

Perceived stress was assessed through the Perceived Stress Scale (PSS), a 10-item questionnaire, for each item a score from 0 to 4 was requested, where 0 stands for Never, 1 almost never, 2 sometimes, 3 quite often, 4 very often. The items assess how people find their lives unpredictable, uncontrollable, or overloaded. The scale also contains a series of direct questions about current levels of perceived stress.

Anxiety symptomatology

The BAI is a self-report tool, composed of 21 items, which allows to evaluate the severity of anxious symptomatology in adults and adolescents.The score attributed to each item is expressed on a Likert scale, from 0 to 3, where 0 stands for "Not at all", 1 "Average", 2 "Moderately", 3 "Severely".

Pain sensation was also evaluated using a question about the maximal pain suffered in the last 3 months, evaluated in a numerical scale from 0 to 10

Emotional functioning

Emotional regulation

Emotional regulation was investigated through a Emotional Regulation Questionnaire (ERQ) and Emotional Suppression (ERS); the questionnaire investigated emotional experience with 10 item. Each item was asked to rate on a Likert scale of 1 to 7, with 1 indicating "strongly disagree" and 7 "strongly agree".

Relational functioning

The quality of the relationship was investigated through the Romance Qualities Scale (RQS), a 22-item scale that represents the five indicators of the quality of the relationship: closeness, conflict, compassion, help and security. The score attributed to each item is expressed on a Likert scale, from 1 to 5, where one stood for "absolutely false" and 5 "represents absolutely true".
